# Doxycycline sclerotherapy of cervical spine aneurysmal bone cysts: single-institution 13-year experience

**DOI:** 10.1007/s00247-022-05328-4

**Published:** 2022-03-19

**Authors:** Megan N. Wong, Leah E. Braswell, James W. Murakami

**Affiliations:** 1 The Ohio State University College of Medicine, Columbus, OH USA; 2 Department of Radiology, Nationwide Children’s Hospital, 700 Children’s Drive, Columbus, OH 43205 USA

**Keywords:** Aneurysmal bone cyst, Cervical spine, Children, Doxycycline, Interventional radiology, Sclerotherapy

## Abstract

**Background:**

Aneurysmal bone cysts (ABCs) are benign, locally aggressive neoplasms that typically affect patients during their first two decades of life. Curettage with or without bone grafting or adjuvants is the current standard treatment; however, other surgical and medical treatments, such as sclerotherapy, have been reported. Treatment options for cervical spine ABCs are more limited because the proximity of ABCs to critical structures leads to greater risk of spontaneous or treatment-related adverse events, including death.

**Objective:**

To retrospectively review all children and young adults with cervical spine ABCs treated with doxycycline sclerotherapy at one referral center to assess its viability as a standalone treatment.

**Materials and methods:**

We retrospectively reviewed the clinical notes and imaging of 16 patients treated with doxycycline sclerotherapy for pathologically proven cervical spine ABCs at our institution between May 2008 and March 2021. All patients underwent image-guided percutaneous doxycycline sclerotherapy to ablate the ABC and stimulate bone formation. We assessed clinical outcomes through chart review and described post-treatment imaging outcomes using modified Neer scoring.

**Results:**

Of the 16 total children and young adults treated, 2 were lost to follow-up, leaving 14 patients with a median age of 14.5 years. Twelve of these 14 patients were successfully treated with doxycycline sclerotherapy for a success rate of 86%. One patient experienced one treatment-related complication (Society of Interventional Radiology [SIR] adverse event classification D), before ultimately being successfully treated. Doxycycline treatment failed in two patients, who then underwent surgical management. Post-treatment imaging of successfully treated cases had a mean modified Neer score of 1.3, whereas post-treatment imaging in failed cases had a mean score of 3.5.

**Conclusion:**

Doxycycline sclerotherapy is a viable standalone treatment for cervical spine ABCs because it is safe and effective while avoiding the morbidity associated with open surgical treatments.

## Introduction

An aneurysmal bone cyst (ABC) is a benign lytic neoplasm that commonly affects patients within their first 20 years of life [1–3]. As expansile lytic fluid-filled tumors covered by thin bone, ABCs are often fragile, resulting in pathological fractures that threaten skeletal stability and function [1, 4]. ABCs can occur in any bone in the body, both within the axial, such as the spine or pelvis, or appendicular skeleton [1]. While most ABCs present in the metaphyses of long bones, they also commonly arise in the spine [1, 4]. ABCs anywhere in the body can be difficult to treat, with variably reported recurrence risks after surgical treatment of up to 30% [1, 2]. Cervical spine ABCs are no different, as is highlighted in the literature, which cites 20–30% recurrence rates for pediatric cervical spine ABCs, regardless of the primary treatment performed [5, 6]. Cervical spine ABCs are especially difficult to treat because they neighbor many critical neurovascular structures and threaten the structural integrity of the neck [5].

Complete surgical resection of an ABC significantly reduces the risk of recurrence, but this approach is often difficult, if not impossible, in the cervical spine, where too aggressive a resection destabilizes the cervical spine and risks spinal cord or nerve root injury and profound blood loss [1, 6, 7]. The surgical literature highlights the danger inherent in removing any type of cervical spine tumor; a review of 110 surgically treated cervical tumors revealed morbidity and mortality rates reaching 42% and 2%, respectively [8]. Additionally, in cases where complete cervical ABC excision is possible, the resultant structural instability usually mandates multilevel vertebral fusion and instrumentation, which is best avoided in physically active and growing children [7].

Because of the difficulties in accomplishing complete cervical spine ABC resection, the current standard of care is some combination of curettage and grafting, with or without adjuvants but often still requiring fusion and instrumentation [4, 9]. Despite being the preferred surgical treatment, one study estimates curettage of cervical spine ABCs to have a 48% local recurrence rate, such that alternative treatment modalities have been sought [6]. Radiotherapy can be an effective treatment option; however, it is usually reserved for targeting inaccessible or recurrent spinal ABCs [1, 10]. Radiation of the cervical spine risks development of myelopathy, injury to adjacent organs, malignant transformation of the lesion or development of secondary malignancies, and osseous growth arrest in pediatric cases [6, 10]. Recently, denosumab, a human monoclonal antibody that inhibits osteoclast stimulation, has been introduced as a potential medical treatment of unresectable ABCs following reports of its use in treating giant cell tumors of bone [11]. Early reports propose denosumab as an adjuvant or a standalone therapy for ABCs [12, 13].

Sclerotherapy has been used successfully to treat ABCs [14–16]. It has been performed with a variety of agents, such as Ethibloc (Ethnor Laboratories/Ethicon, Norderstedt, Germany), polidocanol and a combination of calcitonin and methylprednisolone [4, 5, 14]. Doxycycline is an antibiotic that has anti-tumor properties and stimulates bone growth, suggesting that it would be a good agent for ABC sclerotherapy [15–18]. In 2013, Shiels and Mayerson [15] reported doxycycline sclerotherapy of 21 ABCs that were predominantly appendicular, with a 5% recurrence rate. ABCs that healed after doxycycline sclerotherapy treatment were characterized by (1) the reduction or resolution of lytic areas and fluid-filled cysts and (2) the appearance of new bone growth [16]. Building on previous work with relatively more easily approached appendicular ABCs, we have applied doxycycline sclerotherapy to more challenging lesions in the axial skeleton. In this paper, we report on a subset of ABCs in our single-institution 13-year experience in treating axial ABCs, specifically those in the cervical spine. Our aim in this retrospective review of cases is to show that doxycycline sclerotherapy is a viable first-line standalone therapy for treating cervical spine ABCs.

## Materials and methods

This retrospective study obtained institutional review board approval with the formal informed consent requirement waived. We identified all children and young adults with pathologically proven cervical ABCs treated with doxycycline sclerotherapy at our institution between May 2008 and March 2021 and reviewed their electronic medical records and imaging. For this study, patients were included in the final cohort if they completed the treatment and had both clinical and imaging follow-up or if they began the sclerotherapy treatments but needed to be converted to surgical management. A total of 16 patients with pathologically proven cervical ABCs were treated during this period. Two patients were lost to follow-up immediately after successfully completing treatment and are not included in the final cohort. We retrospectively recorded patient age and gender, lesion location, extent of vertebral involvement at first treatment, presenting symptoms, treatment technique, number of treatments, medication doses, clinical and imaging outcomes, and any complications (Table 1).

**Table 1:** Patient demographics, treatment and outcomes

Patient	Age (yr) Gender (M/F)	Cervical level	Percentage vertebral ring involved (25%, 50%, 75%, 100%)	Presenting symptoms	Prior treatment	Doxycycline doses (mg) each treatment	Other agents injected	Adjunctive treatment	F/U (mo) since last treatment	Modified Neer score on last F/U scan	Clinical outcome	Recurrence	Complication
1	5F	C4	25%	Asymptomatic incidental finding	N/A	90; 60; 100	N/A	N/A	6	1^a^	Asym	N/A	N/A
2	11 M	C4	50% (>2-cm extra-osseous tumor)	Pain and swelling	N/A	250; 300; 240; 140; 150	N/A	N/A	18	1	Asym	Painless regrowth of small cysts 10 months after 4th treat-ment^b^	N/A
3	13F	C3	50%	Radiculopathy	N/A	50; 50; 50; 80	N/A	N/A	51	2^a^	Asym	N/A	N/A
4	18F	C3	75% (>2-cm extra-osseous tumor)	Pain and radiculopathy	N/A	100; 130; 245; 220; 160; 132; 200; 140; 70; 100	Sodium tetradecyl sulfate #1, BVF #1–3^b^	N/A	71	1^a^	Asym	N/A	N/A
5	16 M	C5–7	75% (>2-cm extra-osseous tumor)	Pain and radiculopathy	N/A	100; 300; 300; 160; 200; 200; 200	N/A	N/A	55	1	Asym	N/A	N/A
6	16F	C3–4	50% (>2-cm extra-osseous tumor)	Pain and radiculopathy	Denosumab^b^	260; 160; 200; 300; 300; 300; 260	N/A	Denosumab^b^	24	1^a^	Rare activity-related pain	N/A	N/A
7	11F	C2	50%	Pain	N/A	140; 200; 160; 200	N/A	N/A	56	1	Asym	N/A	N/A
8	3 M	C2–3	75% (>2-cm extra-osseous tumor)	Pain and swelling	Vertebral artery embolization and alcohol sclero-therapy^b^	300; 240; 170	N/A	N/A	58	1	Asym	N/A	N/A
9	14 M	C6	50% (>2-cm extra-osseous tumor)	Pain, radiculopathy and swelling	N/A	300; 300; 300; 300; 250; 200	N/A	N/A	19	2	Asym	N/A	N/A
10	17F	C2	50%	Pain	N/A	120; 200; 200; 100; 80	BVF #1^b^	N/A	45	1	Asym	N/A	N/A
11	24 M	C2	50%	Pain	Bland cement injection^b^	300; 80; 180; 200	N/A	N/A	34	2	Asym	Small residual cysts, pain 26 months after 3rd treat-ment^b^	PICA infarct on first treatment^b^
12	16 M	C6	25%	Pain	N/A	20; 110; 130	N/A	N/A	21	1	Asym	N/A	N/A
13	10F	C3	50%	Pain, swelling	N/A	300; 300	N/A	Surgery^b^	8	4	Limited ROM	N/A	N/A
14	15 M	C3	75% (>2-cm extra-osseous tumor)	Pain	N/A	200; 200	N/A	Surgery^b^	25	3	Limited ROM, chronic pain	N/A	N/A

*Asym* asymptomatic, *BVF* bone void filler, *F* female, *F/U* follow-up, *M* male, *mo* months, *N/A* not applicable, *PICA* posterior inferior cerebellar artery, *ROM* range of motion, *yr* years of age

^a^Consensus score for cases where two reviewers’ individual scores differed

^b^Patients described in text. In patient 4, bone void filler was tricalcium phosphate; in patient 10, bone void filler was demineralized bone matrix and bioactive glass

**Fig. 1:**
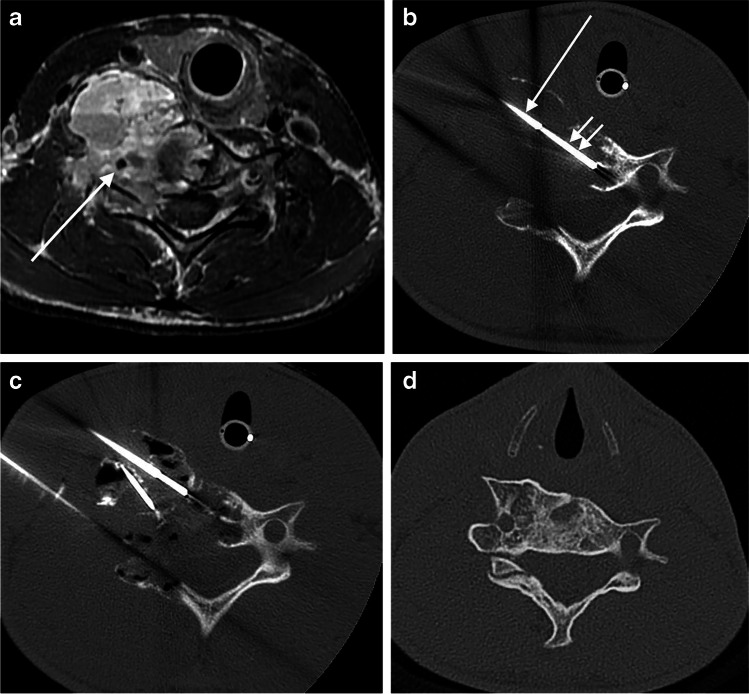
Cross-sectional images from a 14-year-old boy (patient 9) with a C6 aneurysmal bone cyst (ABC). **a** Axial pre-treatment supine T1-W MR image following contrast administration shows a destructive and exophytic multilocular cystic mass replacing the right half of the C6 ring and surrounding the vertebral artery (*arrow*). **b** Axial supine CT image during biopsy and first treatment at the same level as (**a**) shows a 14-gauge (G) guiding needle (*single arrow*) through which passes a 15-G biopsy needle (*double arrows*). **c** Axial supine CT on the same date and at the same level as in (**a** and **b**) shows three separate needles within different portions of the ABC with doxycycline foam (appearing black from air in foam) throughout the different loculations of the lesion. **d** Diagnostic axial CT at same level 3 years after last treatment shows healing of the ABC

Procedures were performed under general anesthesia, after obtaining appropriate procedural informed consent, by three attending pediatric interventional radiologists (L.E.B. with 11 years of post-training experience, J.W.M. with 25 years, and a now deceased third physician, not an author, who had over 30 years of experience). Most of the cases were performed using CT guidance with intermittent CT fluoroscopy on a 64-slice scanner (GE Healthcare Systems, Chicago, IL). Procedures performed more than 10 years ago were done in a similar manner on a 4-slice scanner from the same vendor. One physician used US and fluoroscopic guidance for disease that was exophytic from the vertebrae. In a typical case, CT guidance was used to access the ABC with a mixture of 14-gauge (G) Bonopty needles (AprioMed, Uppsala, Sweden) and 3.5-in. long 18-G spinal needles (BD, Franklin Lakes, NJ). The 14-G needles were needed to acquire biopsies or puncture dense sclerotic bone. During the first treatment, the walls of the cyst were scraped with a 15-G biopsy needle (Bonopty), which was positioned through the 14-G needle to aspirate a mixture of wall lining, septal material and cyst fluid contents for histological evaluation (Fig. 1). Usually, 10–15 mL of bloody fluid and debris is collected for histological evaluation. Often, the lesion is in free communication with the bloodstream and continued aspiration simply yields more and more blood.

After enough needles were placed to ensure adequate coverage of the usually multicystic lesions, less than 1 mL of contrast agent (Optiray 320; Guerbet, Princeton, NJ) diluted 1:1 with saline was injected into each needle to ensure safe placement of all needles. Once imaging showed good dispersal of contrast agent throughout the lesion and no dangerous extravasation, doxycycline biofoam was injected under intermittent CT guidance (Fig. 1). Using the technique previously described by Shiels and Mayerson [15], doxycycline (40 mg/mL in normal saline) was mixed, 1:1, with 25% human serum albumin (Grifols Therapeutics, Clayton, NC) and agitated, 1:1, with air to generate a stable biofoam with a final doxycycline concentration of 10 mg/mL. The biofoam is easily visible and monitored by CT or US because of the air it contains (Fig. 1). All injections employed double-needle injection technique to allow egress of pressure and cyst contents from the needles not being injected [19]. Hand-rolled Surgifoam pledgets (Ethicon, Somerville, NJ) were deployed through the 14-G needles as they were withdrawn to prevent back bleeding from bone puncture sites. Eighteen-gauge needles were simply removed with gentle manual pressure, usually for 2–3 min, at the sites until any bleeding ceased. Procedures were outpatient in nature with several days of pain medication prescribed when needed. Some of the patients were in soft cervical support collars before their procedures, primarily for pain relief. If they were in collars on arrival, they left in the same collars, but no new collars were added to the care of any patients after a procedure.

All patients were treated in a staged manner with treatments every 3 months until the symptoms had resolved and follow-up imaging showed (1) resolution of any fluid-filled cystic areas, (2) that the bone was sufficiently thick to allow normal physical activity and (3) that any remaining small unossified zones were filled with solid (usually fatty or fibrous) tissue rather than fluid (Fig. 1). In the more aggressive cases and at the discretion of the treating interventional radiologist, treatments are performed every 2 months until healing is clearly underway before switching to 3-month intervals. The decision of when to stop treatments can be difficult and nuanced, requiring an accurate assessment of both the images and the clinical situation. Once the last treatment is performed, our standard follow-up strategy is to have the patient imaged yearly with CT or MRI for 5 years, with shorter intervals in select patients.

We evaluated clinical outcomes by chart review and included any residual symptoms or activity restrictions. In addition, we noted and recorded any treatment-related complications. We assessed imaging outcomes from the latest imaging, CT or MRI, follow-up using modified Neer scoring [20]. This scoring system has been adapted from previous studies and was used in this study to rank bone healing from 1 to 4, where a score of 1 indicated a healed lesion that was filled with new bone that might include static, radiolucent area(s) that were less than 1 cm in size. A score of 2 indicated healing with a defect, meaning the bone healed with static, radiolucent area(s) that were <50% of the bone’s diameter but comprised a cortical thickness thought to be sufficient to prevent fracture. A score of 3 indicated persistent cystic area(s) that were >50% of the affected bone’s diameter with thin cortical covering. Even if these residual areas did not increase in size, they continued to restrict patient physical activity and therefore required more treatment. Finally, a score of 4 indicated new cystic areas that were >50% of the affected bone’s diameter with thin cortical covering, necessitating additional treatment.

Neer scoring was independently completed by two investigators (L.E.B and J.W.M), who evaluated the cohort’s most recent follow-up imaging to score the treatment outcomes. If scores differed for a patient, the discrepancy was resolved through consensus review between the same two investigators. Inter-rater agreement was assessed using Cohen kappa (κ) statistic [21].

If there was recurrence, defined as a new or growing cyst within the lesion, during follow-up, doxycycline sclerotherapy was repeated to the satisfaction of the treating specialist (i.e. sufficient osseous healing observed and resolution of clinical symptoms). Successful treatment was defined by the presence of stable osseous healing on imaging (Neer 1 or 2) and the diminution or resolution of clinical symptoms. Treatment failure was defined as significant persistence or enlargement of the cystic regions (Neer 3 or 4), worsening clinical symptoms, and the treating specialist’s clinical concern for potential vertebral collapse and paralysis. Failure indicated the need for open surgical intervention. Our standard practice is to follow all patients clinically and with cross-sectional imaging for approximately 5 years after completion of therapy to monitor for any signs of recurrence. Because this is a review of all previously treated patients, some had 5 years or more of follow-up while some were still in the 5-year surveillance period and therefore had fewer than 5 years of follow-up at the time of this report.

## Results

### Patient cohort

Review of our database identified a total of 16 patients treated with doxycycline at our institution between May 2008 and March 2021. Two patients (ages 17 and 19) were lost to follow-up immediately after successfully completing treatment without complication, leaving 14 patients in the cohort for this report. The patient ages of this cohort ranged from 3 years to 24 years (all patients except one 24-year-old man were 18 years or younger), with a median age of 14.5 years (interquartile range [IQR] of 5 years). Patients underwent 2–10 procedures with a median of 4 (IQR of 2.8). Relevant demographic and clinical details from this study’s cohort are listed in Table 1.

### Interventional treatments and adverse events

All 14 patients in our cohort underwent open surgical or image-guided percutaneous needle biopsies yielding the diagnosis of ABC. All 14 patients were treated with CT-guided doxycycline sclerotherapy. In addition, three of the patients had extraosseous portions of their tumors treated under US and fluoroscopic guidance. Each treatment comprised 20–300 mg doxycycline with a mean dose of 184 mg doxycycline (standard deviation [SD] ± 82 mg). Median follow-up at the time of this report was 29.5 months (IQR of 13.8 months).

Three patients received different treatments at other institutions before coming to our institution for doxycycline sclerotherapy: patient 11 had bone cement without a sclerosant injected in an open surgical procedure after a separate open surgical bone biopsy; patient 6 was treated systemically with Denosumab for 4 months after needle biopsy; and patient 8 underwent vertebral artery coil embolization followed by a single image-guided percutaneous alcohol sclerotherapy session after needle biopsy. All three experienced increased pain and ABC growth after these initial treatments. In patient 6, Denosumab therapy had been ongoing for several months prior to the first doxycycline sclerotherapy session, at which time Denosumab was discontinued. There was robust ABC regrowth with resurgence of pain 4 months later, near the time of the second sclerotherapy session. At this time, after discussion with her referring oncologists, she was restarted on Denosumab, which was tapered and discontinued 6 months after the last sclerotherapy session.

While most of the cases were treated identically by our group with doxycycline as the only active agent, some exceptions deserve comment. Early in our experience, two patients were injected with agents in addition to doxycycline. The first, patient 4, had the first 3 of her 10 doxycycline treatments completed with the addition of Vitoss tricalcium phosphate bone void filler (Stryker, Kalamazoo, MI) into large cystic areas, and 1 treatment with added 3% sodium tetradecyl sulfate (STS) (Mylan Institutional Inc., Rockford, IL) into extraosseous tumor. The second patient, patient 10, had the first of five doxycycline treatments completed with a proprietary bone void filler mixture of bioactive glass and demineralized bone matrix injected to fill a large cystic space.

One patient (patient 11, a 24-year-old man) experienced a single procedure-related complication (SIR adverse event classification D). During the first treatment of the patient’s C2 ABC, the sclerotherapy triggered spasm of the right posterior inferior cerebellar artery (PICA), which was diagnosed with CT angiography, resulting in a PICA distribution cerebellar infarction. This man’s ABC had been treated with open biopsy and cement injection at an outside hospital, which might have decreased the osseous containment of the injected doxycycline. For reasons outside our control, he ended up in an adult hospital in another city where the treating neurosurgeon thought he required a posterior fossa decompression and ventriculoperitoneal shunt placement. In addition, a C0–3 posterior fusion was performed during the decompression. The patient’s C2 ABC, unaddressed at the time of this emergency surgery, was successfully treated by us later with additional doxycycline treatments, injected around the fixation device. This man clinically recovered fully from his cerebellar infarction. There were no deaths in our cohort.

### Clinical response

**Fig. 2:**
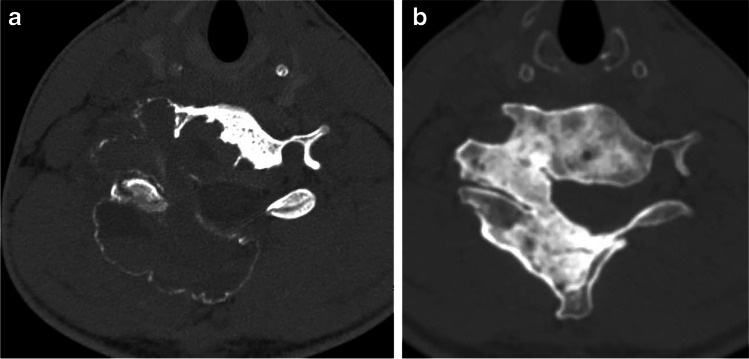
Computed tomography images in a 16-year-old boy (patient 5) with an aneurysmal bone cyst (ABC) involving C5, C6 and C7. **a** Diagnostic axial CT at C6 level before first treatment shows ABC expanding and replacing the entire right half of the vertebral ring at that level. **b** Diagnostic axial CT at same level 5 years after last treatment shows sclerotic bone has replaced the entirety of the ABC. This appearance was similar at all the other levels

Twelve of the 14 patients (86%) were cured of their disease as of the last follow-up, needing no treatments for their ABCs other than the doxycycline sclerotherapy we performed (Figs. 1 and 2). Presenting symptoms of pain, swelling and radiculopathy resolved in all patients except one (patient 6), who had a large lesion involving C3 and C4 and has rare activity-related pain. Two of the 12 successfully treated patients (16%), patients 2 and 11, experienced recurrence during follow-up at 13 months and 30 months after completing initial treatments (one was asymptomatic, and one had recurrent pain). Both were successfully treated with one additional doxycycline sclerotherapy treatment.

**Fig. 3:**
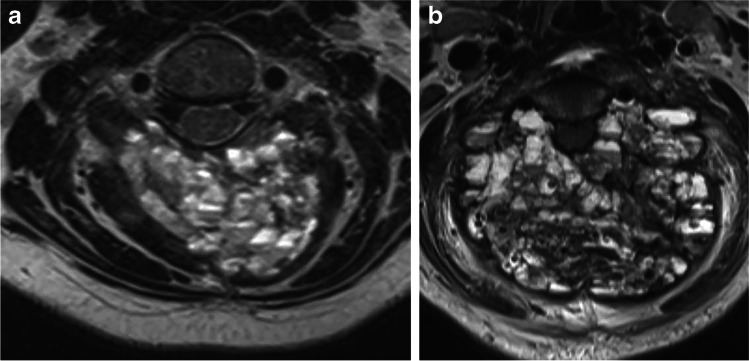
Axial MR images in a 10-year-old girl (patient 13) with an aneurysmal bone cyst (ABC) of the posterior half of the C3 ring. **a** Axial MR image 2 weeks before the first treatment shows expansion and replacement of spinous process and bilateral lamina at C3. More aggressive ABCs tend to have innumerable tiny cysts, as depicted in this case. **b** Axial MR image at the same level 3 months later shows near doubling in size of this very aggressive lesion, now with effacement of the canal

Doxycycline sclerotherapy failed in 2 of the 14 patients (14%). Patients 13 and 14, coincidentally both with C3 spinous process and bilateral laminar disease, experienced ABC enlargement or non-regression and reported worsening clinical symptoms, which resulted in open surgical treatment. Patient 13 (Fig. 3) was treated surgically from a posterior approach with tumor resection and C1–5 fusion, while patient 14 was treated with vertebral artery embolization, preoperative tumor embolization, anterior approach for vertebral body removal, cage insertion and fusion, as well as posterior approach for tumor resection and fusion. The first patient is now pain-free but with limited range of motion and the second struggles with chronic neck pain and limited range of motion, though both were tumor-free at 1- and 3-year follow-ups, respectively.

### Imaging response

Imaging studies for the successfully treated patients all demonstrated healing. Modified Neer scores of treatment outcomes for all 14 patients are in Table 1. The interrater reliability showed moderate agreement (κ=0.55) [22]. Of note, all four interrater scoring discrepancies arose between a score of 1 versus 2, or 3 versus 4. There was 100% interrater agreement between calling a treatment a success (1 or 2) versus a failure (3 or 4). Patients successfully treated with doxycycline sclerotherapy had scores of 1 or 2, with a mean score of 1.3. Imaging of the two failed cases (patients 13 and 14) had modified Neer scores of 3 and 4, respectively, with a mean score of 3.5.

## Discussion

Aneurysmal bone cysts are benign, locally aggressive bone tumors with high rates of recurrence after treatment that usually affect children and adolescents [1, 4, 5]. They most commonly occur as primary lesions in the metaphyses of long bones, though they often also present in the spine [1]. Approximately 70% of ABCs are reported to be primary lesions, while 30% are thought to be secondary to associated pathologies [5]. In approximately 75% of cases there is a balanced chromosomal translocation with involvement of the ubiquitin carboxyl-terminal hydrolase 6 (*USP6*) gene on chromosome 17p13 [4, 22]. This translocation event increases TRE17 production, which increases matrix metallopeptidase (MMP)-9 and MMP-10 activity [4]. These MMPs are responsible for preventing osteoblast maturation and increasing vascular endothelial growth factor (VEGF) release, which leads to increased vascularization that is characteristic of ABCs [4].

Preliminary diagnosis can be achieved via imaging [22, 23]. Biopsies allow for more definitive diagnosis, with specimens marked by fibroproliferative stroma, multinucleated giant cells (osteoclasts), hemosiderin laden macrophages and vascular spaces as hallmarks of ABCs [13, 22]. Further diagnostic certainty can be obtained by assaying for a *USP6* translocation. Needle biopsy of primarily cystic bone lesions is difficult because specimens have only scant cellular elements from the cyst margins.

Cervical spine ABCs are some of the most challenging of ABCs to treat. Complete surgical resection of ABCs with or without adjuvants is associated with the lowest recurrence rates [1, 22]. Practically speaking, this strategy is limited to smaller, more isolated ABCs such as those in the spinous process or a lamina at one level. This is the minority of cases in this report’s cohort where, on average, the ABCs extended 50% of the way around the spinal canal (Table 1). Most successful resections of anything but the simplest of ABCs still result in spinal instability requiring multilevel spinal fusions and instrumentation with resultant lifelong limited neck mobility [7]. Even with complete resections, cervical spine ABCs have an 8% recurrence rate [23]. Rather than complete resection, curettage with or without bone void fillers or adjuvants, coupled with spinal fixation, remains the standard of care despite its 10–60% recurrence rate [1, 4, 9, 23].

Alternative treatments such as radiotherapy or systemic medical treatment with Denosumab have been reported [1, 23, 24]. Despite their favorable initial outcomes, these treatment modalities have limitations. Radiotherapy is typically used for recurrent ABCs or those in hard-to-reach locations because its longer-term effects remain incompletely known but include myelopathy and secondary malignancies [1, 23]. Denosumab, which blocks osteoclast-stimulated bone resorption, risks development of hypocalcemia and rebound hypercalcemia at the start and end of treatment, respectively [13, 25]. Also, insufficient safety data on systemic use of Denosumab in children warrants further caution when considering it for pediatric ABC treatment because it has potential long-term side effects on the immature skeleton away from the ABC [11, 13]. Denosumab is known to affect the physes of a child’s growing skeleton in addition to the ABC, even after relatively short exposures, resulting in dense metaphyseal bands on radiographs during treatment, and there are insufficient data to know what, if any, more permanent physeal injury could occur with longer medication exposure [26]. Last, recurrence risk of the ABC following cessation of Denosumab is also unknown.

The literature cites sclerotherapy with various agents, including Ethibloc, polidocanol, or calcitonin and methylprednisolone, as a potential treatment option. Ethibloc is no longer available, polidocanol has not been approved for use in the United States, and methylprednisolone can worsen lesions [1, 4, 15]. A recent report cited success in adding transarterial embolotherapy to sclerotherapy for treatment of unresectable or recurrent ABCs [27]. Our only institutional experience with embolotherapy in ABC management in the spine is as a preoperative measure prior to excision of extraosseous disease related to a massive lumbar spine ABC. Also, while embolotherapy has been shown to be a useful adjunct to diminish intraoperative bleeding, it has not significantly reduced recurrence risk following the surgery [23]. Embolotherapy of cervical spine ABCs would add significant risks and challenges to sclerotherapy alone, with questionable gains over what we have demonstrated in this cohort.

Doxycycline has been presented as a sclerotherapy agent for spinal ABC treatment [22, 23, 28]. Doxycycline enhances bone formation by osteoblasts and inhibits MMPs, angiogenesis and osteoclast bone resorption [28]. Its effects should therefore counter some of those resulting from increased TRE17 production inherent in ABC pathophysiology [4, 28]. Prior studies have demonstrated its success as a sclerotherapy agent treating primarily appendicular ABCs, with complication rates <5% and recurrence rates between 6% and 11% [15, 16]. Most patients in those studies were clinically asymptomatic after doxycycline treatment. More recently, studies have assessed the success of doxycycline sclerotherapy as a treatment for spine ABCs [22, 28]. Although these studies comprised relatively few patients, the authors concluded that doxycycline sclerotherapy was a viable treatment for ABCs in difficult locations [22, 28]. Our study supports this conclusion with an 86% cure rate in treatment of 14 cervical spine ABCs using image-guided doxycycline sclerotherapy.

Sclerotherapy of spinal ABCs, particularly cervical spine ABCs, requires a higher level of attention to technical detail than most extremity ABCs. Targeting the many cystic areas in the ABCs requires accurate needle placement and sclerosant injection to avoid non-target tissue injury given a higher density of sensitive tissues near cervical spine ABCs than in most extremity ABCs (Fig. 1). We find that double-needle technique is necessary in all ABC treatments to prevent pressure build-up within the ABC and leakage into adjacent tissues. Also, test injections with dilute contrast agent prior to sclerosant injections can give a preview of where the sclerosant will go once injected. There is a case report journaling the death of a child after Ethibloc injection into a C2 ABC using single-needle technique [29]; the authors postulated there was retrograde flow of the sclerosant through a feeding vessel to the vertebral arteries with resultant vertebrobasilar embolization and brainstem infarction.

Certainly, the risk of sclerotherapy in cervical spine ABCs is higher than in extremity ABCs, but the reward for the patient is also higher because successful sclerotherapy obviates morbidity and mortality risks associated with open surgical procedures on the cervical spine. Cervical spine ABCs require more intensive clinical decision-making than extremity ABCs before, during and after treatment. The treating physician needs to be willing to move on to surgical referral for tumor removal and operative fixation in cervical spine ABCs if it appears the treatment is not working or working too slowly and leaving the patient at risk of collapse, paralysis or death. These nuances of cervical spine ABCs with their higher risk-to-reward ratios than many other ABCs is why we present only cervical spine ABCs in this report. We hope that in later reports we can expound on other neuroaxis ABCs we have treated in the skull, thoracic spine, lumbar spine and sacrum.

In this study, 12 of 14 patients were successfully treated with doxycycline sclerotherapy as a standalone therapy. All except one successfully treated patients were asymptomatic at last clinical follow-up. Using doxycycline sclerotherapy, we successfully treated three patients who received prior treatment at outside institutions. Treating spine ABCs can be a much greater challenge when working on a recurrent tumor in a postoperative bed, especially if one must work around fixation devices such as plates, screws, cages, cement and rods, which make targeting difficult on any image-guided procedure.

Of the 12 successful cases we present, 2 patients suffered cyst recurrence identified on follow-up imaging and these were treated successfully in each patient with one additional doxycycline treatment. This highlights the need to follow these patients with imaging for years after successful treatment. Our protocol is to follow all our patients for 5 years after treatment to search for any recurrence of cystic bone destruction. While it is not our routine clinical practice to score our follow-up imaging using numerical modified Neer scoring, we did use it in this paper to give a more precise description of the outcomes. The two reviewers scored all successful cases as Neer 1 or 2 and both failures as Neer 3 or 4, which fits with our more qualitative assessment of success in these patients, which is always a combination of the imaging and any clinical symptoms. Predicting which patients need more sclerotherapy and which can be observed is difficult, and other authors have also presented a grading system that correlates with their outcomes [30]; the radiographic and CT components of their grading system for ABC healing after sclerotherapy were nearly identical to those in the Neer system we used, though theirs had five grades and ours had four. More important, the other authors found, as we do, that a combined radiographic analysis of healing together with clinical parameters such as persistent pain are needed to predict who would benefit from more sclerotherapy.

Doxycycline sclerotherapy failed in treating 2 of our 14 patients. While we have no definitive explanation for these failures it has been our experience with many other ABCs in all areas of the axial and appendicular skeleton that lesions that appear particularly aggressive by imaging are often just that. Lesions that are very expansile, have innumerable sub-centimeter cysts (Fig. 3), have many fluid-fluid levels indicating repeated episodes of bleeding, and cause greater than average pain from rapid growth and fracture tend to be resistant to therapy. These lesions tend to have brisk bleeding for both the interventional radiologist and the surgeon. For this reason, our practice when working with these lesions is to decrease the interval of serial therapy from 3 months to 2 months in hopes of more effective treatment. Despite this approach, with these two patients one of the lesions did not respond and one nearly doubled in size (Fig. 3) after the first treatment and they were referred for surgery.

The primary limitation of this study derives from its being retrospective, such that all patients were not treated precisely the same, though most procedures performed in the 14 patients, 59/65 (91%), were completed with the same technique and injected agents. The exceptions were detailed earlier in the paper. Three of the patients had failed treatments of different kinds at other hospitals prior to coming to us. The contribution of those prior treatments to the final outcomes cannot be fully known, but in all three cases the patients were ultimately successfully managed with doxycycline sclerotherapy. This treatment variability inherent in a retrospective series such as this one could pollute the data, but we do not believe it substantively alters the conclusion of this paper. Of the 14 total patients in this reported cohort, 9 came for treatment from out-of-town, with most coming from out-of-state or -country. This made obtaining follow-up sometimes difficult, leading to 2 early patients being lost to follow-up immediately following successful completion of doxycycline therapy. Over time we have learned to be more vigilant in getting imaging and clinical follow-up and we hope to soon start a prospective registry to track all ABC patients’ experiences during treatment and follow-up.

## Conclusion

Image-guided doxycycline sclerotherapy is a safe and effective therapy for cervical spine ABCs and should be considered a first-line standalone treatment option.
